# Convolutional-LSTM approach for temporal catch hotspots (CATCH): an AI-driven model for spatiotemporal forecasting of fisheries catch probability densities

**DOI:** 10.1093/biomethods/bpaf045

**Published:** 2025-06-02

**Authors:** Altair Agmata, Svanur Guðmundsson

**Affiliations:** Bláa Hagkerfið ehf., Skútuvogi 3, Reykjavík 104, Iceland; Bláa Hagkerfið ehf., Skútuvogi 3, Reykjavík 104, Iceland

**Keywords:** artificial intelligence, deep learning, convolutional LSTM, fisheries forecasting, spatiotemporal dynamics

## Abstract

Efficient fisheries management is crucial for sustaining both marine ecosystems and the economies that heavily depend on them, such as Iceland. Current fishing practices involve decisions informed by a combination of personal experience, current data on environmental and oceanographic conditions, reports from other captains, and target species within the constraints of the fishing quota. However, the intricate spatiotemporal dynamics of fish behaviour make it difficult to predict fish stock distributions. Despite technological breakthroughs in fishing vessel data collection, much of the decision-making still relies heavily on subjective judgement, highlighting the need for more robust, data-driven predictive methods. This paper presents CATCH, a convolutional long short-term memory neural network model that forecasts fish stock probability densities over time and space in Icelandic waters to support operational planning and adaptive strategy in fisheries. The framework represents the first utilization of large-scale Icelandic fishing fleet data integrating multidimensional inputs, particularly depth, bottom temperature, salinity, dissolved oxygen and catch data, to produce accurate, multivariate forecasts. The model achieves favourable performance with average RMSE, MAE, WD, and SSI of 4.71 × 10^−3^, 1.16 × 10^−3^, 0.94 × 10^−3^, and 0.955, respectively, for cod, while 6.13 × 10^−3^, 1.25 × 10^−3^, 1.04 × 10^−3^, and 0.949, respectively, across other target species (haddock, saithe, golden redfish, and Greenland halibut). Additionally, Syrjala’s test yielded nonsignificant *P*-values (*P* > .05) in most cases across lags and forecast horizons, indicating that the predicted and observed distributions are statistically indistinguishable. Its promising results suggest deep learning models have the potential to optimize fisheries operations, enhance sustainability, and support data-driven decision-making.

## Introduction

Efficient fishing practices are essential for maximizing the economic benefits derived from marine resources while environmental sustainability is safeguarded. This ensures the long-term preservation of both the ecosystem and economic livelihoods of nations heavily reliant on the fishing industry. Iceland, in particular, is a country where the fishing sector plays a pivotal role in its economy and is widely believed to be the country’s single most important industry [1]. In fact, current statistics estimate that the industry contributes ∼38% of the nation’s total export revenues in 2023, with the total export from fisheries valued at 359 billion ISK in 2023 [2, 3]. The economic significance of the fisheries industry thus underscores the importance of accurate fish stock management strategies. Icelandic waters, influenced by the dynamic North Atlantic currents, are home to commercially significant species, including *Gadus morhua* (Atlantic cod), *Melanogrammus aeglefinus* (haddock), *Reinhardtius hippoglossoides* (Greenland halibut), *Sebastes marinus* (golden redfish), and *Pollachius virens* (saithe). These species, among others, are essential to the economy, with Atlantic cod being one of the most valuable due to its high demand globally and its indispensable role in the ecosystem [2, 4, 5].

In line with the significant role that fisheries play in the economy, the Icelandic fishing industry has evolved to become one of the most technologically advanced of its kind in the world. Over the years, technological innovations have enabled companies to optimize their operations significantly throughout the value chain. These advancements facilitated the collection of massive and diverse datasets that include information on fishing time, location, sea temperature, depth, and even on catch volume and composition. Currently, skippers gain insights about where and when to fish by manually reviewing and sharing their ships’ data and combining it with their personal experiences, environmental and oceanographic conditions, and weather forecasts. However, inherent uncertainties in the fishing process limit skippers’ predictive capabilities, which rely heavily on subjective judgment. As a result, there is a pressing need for an accurate prediction method that guides fishermen towards most efficient and environmentally sustainable fishing at all times [6]. While the current data collected is abundant and rich, existing methods fall short of leveraging its full potential because they lack the necessary analytical capabilities to extract comprehensive predictive insights.

To address these challenges, artificial intelligence (AI) offers promising new technologies, particularly deep learning models, for enhancing catch efficiency while minimizing ecological impact in the operation of fishing fleets [7–9]. Neural networks, the foundation of deep learning, are models inspired by the biological brain’s structure and can uncover complex, nonlinear relationships within the data. They are composed of simple, nonlinear modules (neurons) that perform level-wise transformations of increasing abstraction, allowing very complex functions to be learned given enough combinations of such transformations [10]. It is in recent times that AI became the more appealing choice compared to conventional statistical and classical machine learning methods because it can automatically learn nonlinear representations of complex-structured data without relying on hand-crafted features or prior domain knowledge [10, 11]. In contrast, conventional statistical and machine-learning techniques were limited in their ability to process natural data in their raw form, requiring rigorous feature engineering along with considerable domain expertise. This advantage has driven the rise of AI applications like ChatGPT in the natural language processing domain, where the model learns directly from raw training data without needing rules in language such as grammar and syntax to be explicitly specified [12–15].

**Figure 1.**
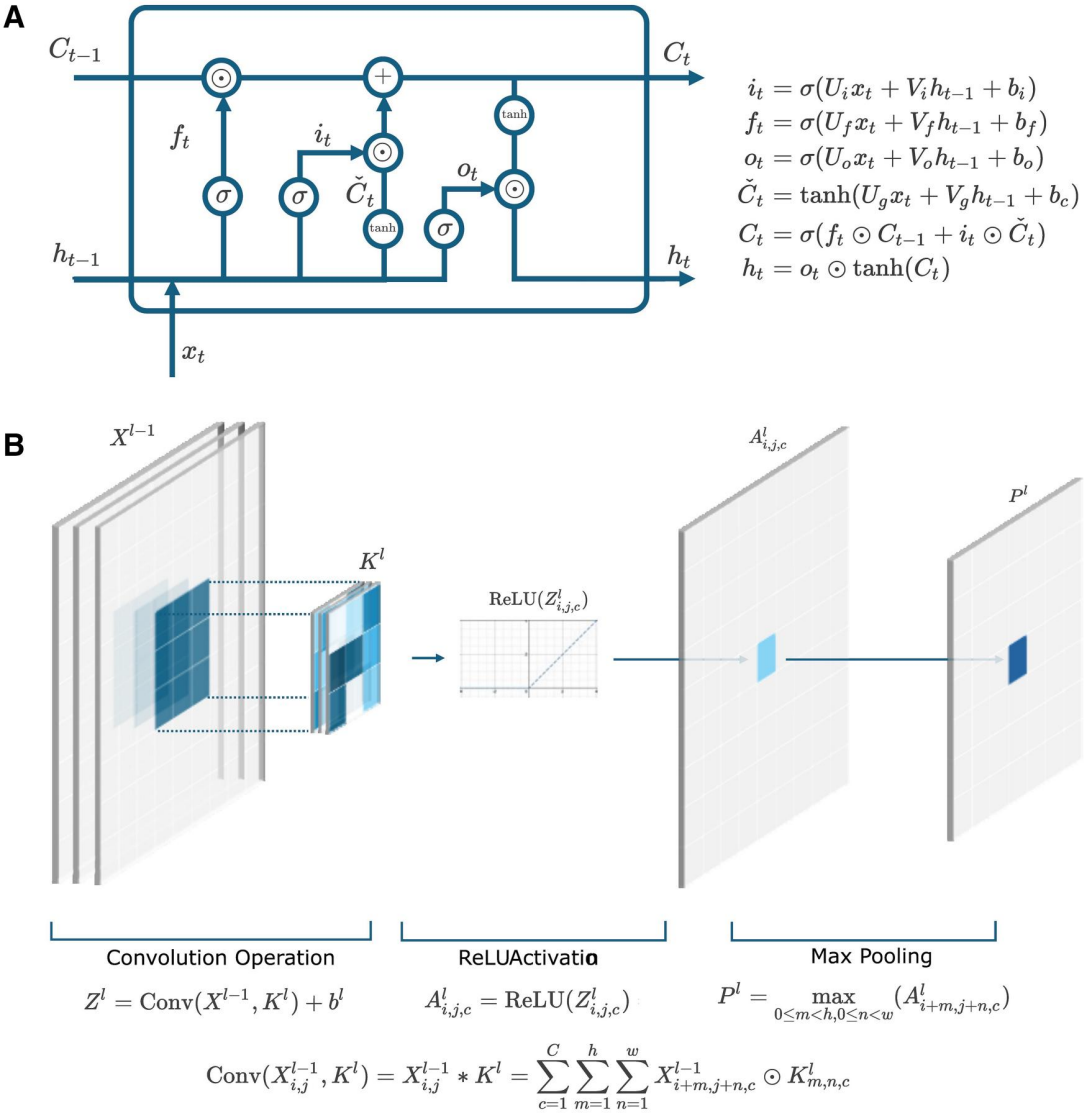
An illustration of the common implementation scheme of (**A**) Long Short-Term Memory (LSTM) neural network [16] and (**B**) Convolutional Neural Network (CNN) [10, 43] along with their corresponding equations.

The spatiotemporal nature of fisheries data requires an architecture that handles both the patterns through time in various temporal scales and spatial relationships across multiple geographical areas. For each of these two tasks, long short-term memory networks (LSTM; Fig. 1A) and convolutional neural networks (CNN; Fig. 1B), respectively, are particularly well-suited. LSTM networks, a special type of recurrent neural networks (RNN), are designed to retain information over long sequences of data and thus highly effective for time-series forecasting use cases. The term ‘long short-term memory’ refers to the short-term memory lasting thousands of timesteps that the LSTM architecture aims to provide for RNNs, giving it the ability to analyse long-term sequence dependencies [16]. It also addresses the common vanishing/exploding gradient problem common with RNNs by keeping track of a separate ‘cell state’ that changes with each iteration [17, 18]. Due to these advantages, LSTM networks are one of the most popular models of choice for sequence-type datasets and have been successfully applied in multiple domains such as finance [19–21], manufacturing [22–24], healthcare [25, 26], meteorology [27–30], and fisheries [7–9]. Meanwhile, CNNs excel at capturing spatial relationships within the data, making them ideal for analysing geographical distributions of fish populations. As the name implies, most of the heavy lifting in the feature extraction process within a CNN comes from the convolution operation that happens within each convolutional layer. Within this layer, local conjunctions of features from preceding layers are detected through discrete convolution between learnable kernel filters and the input/intermediate data resulting in the extraction of invariant features such as edges, motifs, parts, and objects in an image [10]. These features are then used downstream for classification, typically through a shallow or deep dense neural network. The architecture has seen great success in numerous applications where images are the main input data format [31–34] or when data can loosely be transformed into image-like representation [24, 35–37].

An AI architecture that combines the strengths of both CNN’s and LSTM’s thus makes the most sense when tackling the complexity of spatiotemporal fisheries data, particularly when applied to forecasting. Incidentally, such an architecture that literally combines both such frameworks, called Convolutional-LSTM, exists and was first applied to precipitation forecasting applications [38]. It has also become an increasingly popular choice and proven effective in applications involving data with both temporal sequences and spatially organized two-dimensional arrays across a variety of fields [39–42]. This study thus introduces CATCH (Convolutional-LSTM Approach for Temporal Catch Hotspots), which is an AI model with the goal of producing accurate and timely predictions of fish stock probability densities across space and time in the Icelandic waters. This paper is the first in literature to discuss the use of a deep learning model such as convolutional-LSTM to forecast spatiotemporal fisheries dynamics. This is also the first time that the previously unexplored data collected directly from the Icelandic fishing fleet will be utilized at such a computing scale. Notably, the core objective of this study is to validate the predictive capability of the framework, irrespective of the ecological or fisheries-specific context. This focus is essential because the catch data does not directly capture the biological characteristics of the fish, and external biases such as fishing behaviour remain intertwined within the dataset. In Icelandic waters, where environmental variability and fishing pressure can shift rapidly, these AI-driven models open paths for the development of powerful tools for skippers and fisheries operators, to provide them with valuable insights for their operations. Our model integrates multidimensional spatiotemporal data—including temperature, depth, salinity, dissolved oxygen, and catch per unit effort (CPUE) probability densities—to generate outputs that help optimize fisheries operations, potentially enhancing profitability and long-term sustainability of Iceland’s fishing industry.

## Methodology

### Training dataset

The raw data used in this study was a proprietary dataset obtained from various fishing vessels operated by multiple Icelandic fishing companies. These data, encompassing species information, fishing gear, geographical data, vessel information, and environmental parameters, were retrieved using SQL queries to an Amazon Web Services (AWS) Redshift database where the data is stored. Due to the proprietary nature of the data, we are unable to disclose the full dataset or make it public. Additionally, biogeochemical data used as supplementary features in training the model were obtained through the E.U. Copernicus Marine Services [44, 45].

Before the data was used for model training, the retrieved raw data was first filtered and preprocessed to reduce the complexity of the learning task and make it appropriate to produce the insights and predictions expected of the model. The series of filtering steps taken is enumerated as follows:

1. *Limiting to only bottom trawling (Botnvarpa) as fishing gear type*. Since the main metric used to gain prediction insights on fisheries population is CPUE, this filtering was done to standardize the effort parameter from all observations as different fishing gears have varying catching efficiencies. Bottom trawlers in the dataset had the greatest number of observations and also has the least efficiency variance within the dataset, thus making it the best choice for effort standardization among different fishing gear types.
2. *Filtering data to 2008 onwards.* The filtering was done to eliminate sampling bias from sparse observations through time as was observed in the data before 2008. Data quality before the cut-off was extremely low and may lead to skewed patterns when used in downstream training.

**Figure 2.**
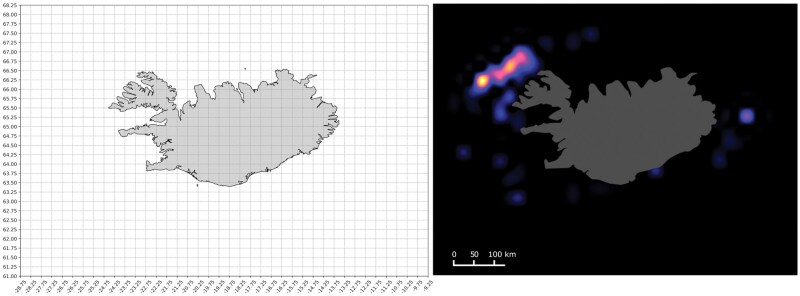
Spatial aggregation of the training dataset showing the binning grid across the Icelandic waters used in the study (left) and the sample snapshot of aggregated CPUE probability distribution observation with cubic spline interpolation for Cod in November 2012 (right).

The filtering process resulted in a dataset with 274 521 spatiotemporal observations spanning across five species: *Gadus morhua* (Atlantic cod), *Melanogrammus aeglefinus* (haddock), *Reinhardtius hippoglossoides* (Greenland halibut), *Sebastes marinus* (golden redfish), and *Pollachius virens* (saithe). The accuracy of species identification within this dataset is ensured by the fact that Icelandic fisheries are strictly regulated according to national mandates such as Regulation No. 745/2016 and the performance agreements of Fiskistofa [46–48]. Fishing vessels are legally required to report catches by species with high fidelity, enabling reliable species-level labelling in the underlying data sources. This dataset is then preprocessed to prepare it for the model training process:

1. *Feature selection.* Features used for training and prediction were limited to five variables: CPUE (kilograms per minute), bottom temperature (Celsius), depth (meters), dissolved oxygen (mmol/m^3^), and salinity (practical salinity unit). The temperature, depth, dissolved oxygen, and salinity variables were selected, because of their hypothesized impact and data quality. CPUE on the other hand, was obtained by dividing the weight of the catch (kg) with the tow duration (min) for each observation. This normalized the effect of tow duration per catch, thus showing a glimpse of the fish population density at a specific time and area of the catch record. All the ships included in the data have similar average speeds and tow coverage, so these factors were not included in the CPUE calculation.
2. *Binning observations across space and time.* The granularity of the observations was reduced through binning and aggregation. Binning was done with respect to space and time, imposing the assumption of local similarity within each bin. In this study, spatial binning was performed using increments of 0.5° longitude by 0.25° latitude ranging from −28.75° to −9.25° longitude and 61° to 68.25° latitude (Fig. 2). This translates to ∼24 km × 28 km around this sampling range. This resolution was chosen to match the typical spatial scale of bottom trawling activity, which ranges from 5.4 km to 63 km, based on FAO gear operation estimates [49]. The finer resolution along the longitude axis accounts for the convergence of meridians at higher latitudes, thereby maintaining consistent kilometre-scale precision across both dimensions. Temporally, data was binned per month, reflecting operational timescales in fisheries and capturing seasonal trends without over-fragmenting the data. Binning was set as mentioned to avoid information loss from using excessively large bins and to prevent proliferation of numerous uninformative small bins. Aggregation per bin was done by getting the mean of each group.
3. *Converting features to probability distributions.* This was done by normalizing each binned spatiotemporal observation for each feature to fractions of unity. Mathematically, it can be expressed as:
  (1) $${\hat{X}}_{s,h,w,c}\;=\;\;\frac{X_{s,h,w,c}}{\sum_{h}\sum_{w}X_{s,h,\;w,c}}\in R^{S\times H\times W\times C}$$
  where X and $\hat{X}$ represent the raw and probability density tensors, respectively, of size *S* × *H* × *W* × *C* for each sample observation *s*, latitude *h*, longitude *w*, and feature *c*. Converting the raw feature values to probability densities offers several advantages with respect to forecast modelling, such as enforcing stationarity by normalizing temporal and spatial variability and focusing on relative distribution dynamics, among others. In the context of CPUE, this normalization means that each value can be interpreted as the fraction of the total catch per unit effort attributed to a specific spatial bin, capturing the relative likelihood of a catch occurring there. This transformation shifts the emphasis from absolute CPUE values to their spatial distribution pattern, thereby mitigating the influence of varying fishing effort and external biases on the predictive model.
4. *Expanding the input tensor to time-lag dimensions.* A common technique used for temporal forecasting predictions to teach the model temporal relationships is by expanding the input tensor to time-lagged version of itself. In this study, time-lag values (*L*) of 3, 6, 12, 16, and 24 months were tested representing various spans of temporal variabilities to be considered per prediction resulting in a final 5D input tensor $\bar{X}$ ∈ ℝ^*S*^^×^^*L*^^×^^*H*^^×^^*W*^^×^^*C*^. The lag size *L* determines how many previous months of data are provided as input to the model for each prediction. This temporal window acts as the model’s context length, allowing it to learn sequential dependencies in the data. Smaller lag sizes help the model focus on short-term fluctuations, while larger lag sizes provide a broader historical perspective to capture longer term temporal patterns. Testing multiple lag values enables the evaluation of the model’s sensitivity to different temporal dynamics present in the fisheries dataset.

### Model architecture

**Figure 3.**
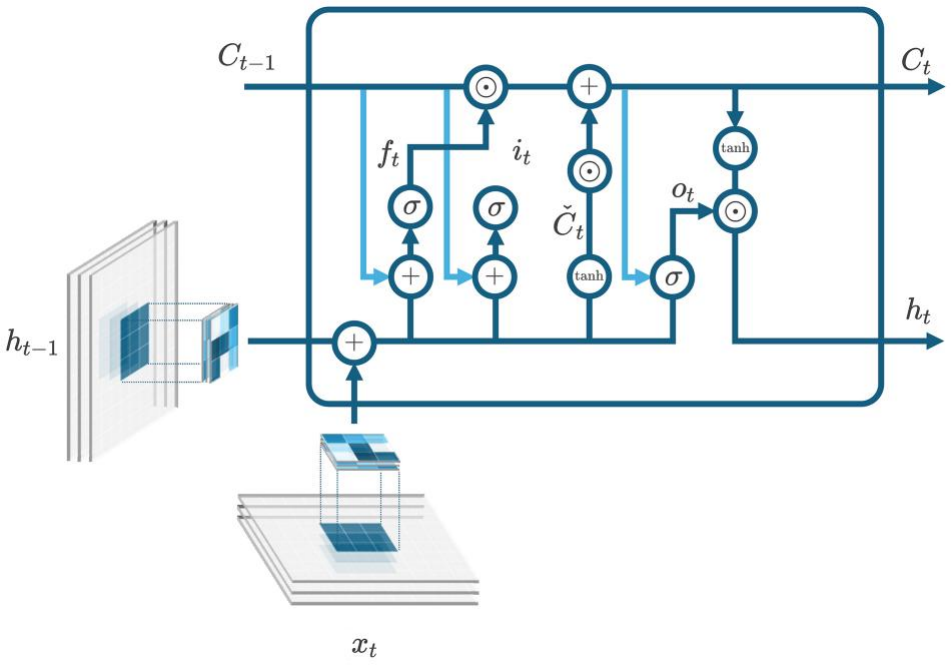
Convolutional-LSTM (ConvLSTM) neural network cell used in the study based on Shi *et al*. [38].

The core component used in the CATCH model was a convolutional-LSTM (Fig. 3) [38] neural network cell designed to capture both spatial and temporal dependencies in fisheries data. The architecture of the model per layer is mathematically expressed as:

(2) $$\begin{array}{c} i_{t}\;=\;\sigma (U_{i}\;*\;x_{t}+V_{i}\;*\;h_{t-1}+W_{i}\circ C_{t-1}+b_{i})\\ f_{t}\;=\;\sigma (U_{f}\;*\;x_{t}+V_{f}\;*\;h_{t-1}+W_{f}\circ C_{t-1}+b_{f})\\ \begin{array}{c} o_{t}\;=\;\sigma (U_{o}\;*\;x_{t}+V_{o}\;*\;h_{t-1}+W_{o}\circ C_{t-1}+b_{o})\\ \hat{C_{t}}\;=\;\tanh\left(U_{g}\;*\;x_{t}+V_{g}\;*\;h_{t-1}+b_{c}\right)\\ \begin{array}{c} C_{t}\;=\;f_{t}\circ C_{t-1}+i_{t}\circ \hat{C_{t}}\\ h_{t}\;=\;o_{t}\circ \tanh\left(C_{t}\right) \end{array} \end{array} \end{array}$$

where ‘◦’ represents the Hadamard product and ‘∗’ the convolutional operation between the trainable kernel weights and an input image described by equation (3) in the case of convolution on input image *x*. In this architecture, the convolution operator is applied in the hidden and input states of LSTM.

(3) $${\left(U\;*\;x\right)}_{h,w}\;=\;\text{Conv}(U,x_{h,w})\;=\;\sum_{c\;=\;1}^{C}\sum_{m\;=\;1}^{K_{h}}\sum_{n\;=\;1}^{K_{w}}U_{m,n,c}\circ x_{h+m-1,w+n-1,c}$$

**Figure 4.**
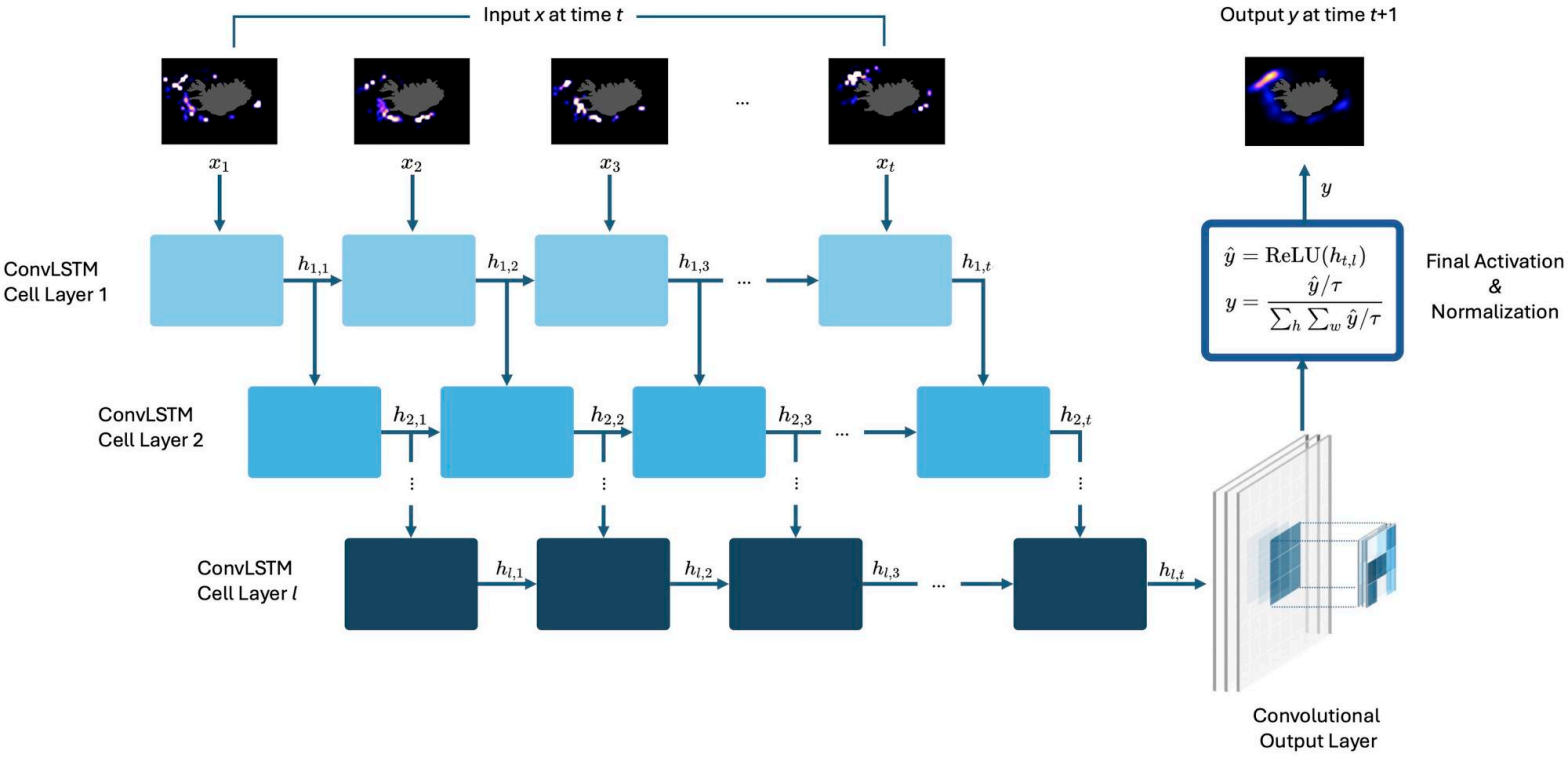
CATCH forecasting model architecture.

Using the core component as building blocks, a multilayer model architecture is built representing different layers of data abstraction and expression. Figure 4 shows the implemented architecture for the study wherein each layer’s hidden state is used successively as input towards succeeding ConvLSTM layers. In these layers, expressivity of the data is increased by expanding the feature dimensionality *C* with the use of *F* kernel filters of size *K* with *F > C*. At the last layer, the last hidden state is extracted and passed through the output convolutional layer with *F* 3×3 kernel filters, compressing the output back to its original dimension *C*. Finally, the convolution output passes through a ReLU activation function to zero out negative probability predictions and the resulting logit *ŷ* is normalized in a similar way with equation (1):

(4) $$y_{b,t,h,w,c}\;=\;\frac{{\hat{y}}_{b,t,h,w,c}/\tau }{\sum_{H}\sum_{W}{\hat{y}}_{b,t,h,w,c}/\tau }\;\in R^{B\times L\times H\times W\times C}\;$$

where *τ* is a temperature hyperparameter that regulates the sharpness of the output probability distributions—i.e. lower values of *τ* produce more peaked (low-entropy) distributions, while higher values result in smoother (high-entropy) distributions.

### Training and hyperparameter tuning

The model is trained through backpropagation through time (BPTT) [50] by minimizing a weighted binary cross-entropy (BCE) loss, which is used to prioritize the accuracy towards the CPUE probability prediction. For each feature *c*, the BCE loss for a single element is given by:

(5) $${\mathrm{BCE}}_{c}(y_{\mathrm{obs}},\;y_{\mathrm{pred}})\;=\;-\frac{1}{N}\sum y_{c,\;\mathrm{obs}}\log y_{c,\;\mathrm{pred}}+(1-y_{c,\;\mathrm{obs}})\log\left({1-y}_{c,\;\mathrm{pred}}\right)$$

 

(6) $$\mathrm{Weighted}\;\mathrm{BCE}\;\mathrm{Loss}\;=\;\sum_{c}w_{c}{\mathrm{BCE}}_{c}$$

In this study, the weights *w_c_* for each feature used are 0.3 for CPUE probability and 0.175 for the rest of the four features.

The model was trained using data of varying lag size, and thus model context window, to gain prediction insights from variations attributed to short-term and long-term patterns. Specifically, context sizes of 3, 6, 12, 18, and 24 months backward were used in the study but only focusing on cod (*G. morhua*) first as this is the species with the greatest number of observations within the dataset. The context length with the best results is then used to train and predict across all the five species. Each dataset was partitioned into training, validation, and test sets. The training set includes data from January 2008 to May 2022, while the validation and test sets each comprise the subsequent 12-month periods: June 2022 to May 2023, and June 2023 to May 2024, respectively. Hyperparameters for each of the training run were obtained automatically using Bayesian optimization through the KerasTuner library across 100 trials with the objective of minimizing the validation loss. The hyperparameter values/ranges used in the hypertuning process were summarized in Table 1. Decisions about the ranges and fixed values were made based on prior experiments, computational efficiency, and available compute resources.

**Table 1.** Hyperparameter values/ranges explored in the study.

Hyperparameter	Value/range
Number of filters (*F*)	[3, 5]
Kernel size (*K*)	9 × 9
Number of ConvLSTM layers (*L*)	[2, 3]
Dropout in ConvLSTM layers	[0.2, 0.3]
Temperature (*τ*)	[0.1, 0.3]
Max epoch (*E*)	200
Batch size (*B*)	16
Learning rate (*R*)	[1 × 10^−5^, 1 × 10^−3^]

### Model evaluation

Predictions are evaluated based on performance with regard to the model’s one-at-a-time (OAT) and recursive forecast. OAT prediction refers to predicting only one month at a time and that at every timestep, the model uses an input comprised purely of observed instances. In contrast, recursive forecast is done through generation of sequential predictions for a specified forecast length by iteratively using the model’s previous predictions as inputs for subsequent predictions, creating a chain of forecasts over time while maintaining a consistent input size through slicing. Such predictions are evaluated against the testing data using metrics summarized in Table 2.

**Table 2.** List of metrics used to evaluate model performances and their corresponding mathematical formulations.

Metric	Formulation	
Root mean squared error (RMSE)	$\frac{1}{T}\sum_{t=1}^{T}\sqrt{\frac{1}{N}\sum_{i=1}^{N}{\left(y_{\text{obs},t,i}-y_{\text{pred},t,i}\right)}^{2}}$	(7)
Mean absolute error (MAE)	$\frac{1}{T}\sum_{t=1}^{T}\frac{1}{N}\sum_{i=1}^{N}\left|y_{\text{obs},t,i}-y_{\text{pred},t,i}\right|$	(8)
Wasserstein distance (WD)	$\int_{-\infty }^{\infty }|{\mathrm{CDF}}_{\mathrm{obs}}(x)-{\mathrm{CDF}}_{\mathrm{pred}}(x)|dx$	(9)
Structural similarity index (SSI) (51)	$\frac{\left(2\mu_{y_{\text{obs}}}\mu_{y_{\text{pred}}}+C_{1})(2\sigma_{y_{\text{obs}},y_{\text{pred}}}+C_{2}\right)}{\left(\mu_{y_{\text{obs}}}^{2}+\mu_{y_{\text{pred}}}^{2}+C_{1})(\sigma_{y_{\text{obs}}}^{2}+\sigma_{y_{\text{pred}}}^{2}+C_{2}\right)}$	(10)

RMSE and MAE are standard regression metrics that measure error with varying sensitivity to outliers. Relative fit metrics such as percent error (MAPE) are unsuitable in this context because the response variable frequently contains zero or near-zero values, which can lead to disproportionately large or even infinite percent errors. Although the problem could be treated as a regression task, both the input data and model output are interpreted as probability densities. Consequently, the study employs a suite of evaluation metrics, each offering a distinct mathematical perspective on performance. In particular, the Wasserstein distance (WD) is used to quantify the optimal transport cost required to transform one distribution into another [51] approaching the evaluation as comparison between probability density distributions. Additionally, Structural Similarity Index (SSI) is incorporated, as it is widely used in computer vision to assess differences in intensity, contrast, and structural covariance between images [52], thereby providing a measure of structural consistency across spatiotemporal frames.

In addition to conventional error metrics, Syrjala’s test [53] was implemented to evaluate whether the predicted and observed spatial distributions are statistically distinguishable. Syrjala’s test compares two spatial probability distributions using the discrete analogue of Cramér–von Mises criterion, which computes the squared differences between cumulative distributions over a spatial grid as the test statistic:

(11) $$\Psi \;=\;\sum_{k\;=\;1}^{K}{\left[\Gamma_{1}(x_{k},y_{k})-\Gamma_{1}(x_{k},y_{k})\right]}^{2}$$

In this study, Syrjala’s test is used to test the null hypothesis (*H*_0_) that the predicted and observed distributions are not significantly different—i.e. they may originate from the same underlying distribution. The alternative hypothesis (*Hₐ*) posits that the distributions are significantly different. The test was implemented through Monte Carlo permutation testing, randomly shuffling spatial labels between distributions across 10 000 iterations to generate the null distribution of the test statistic and corresponding empirical *P*-values per comparison. The test was performed across different forecast horizons and lag values (*T*).

## Results

**Figure 5.**
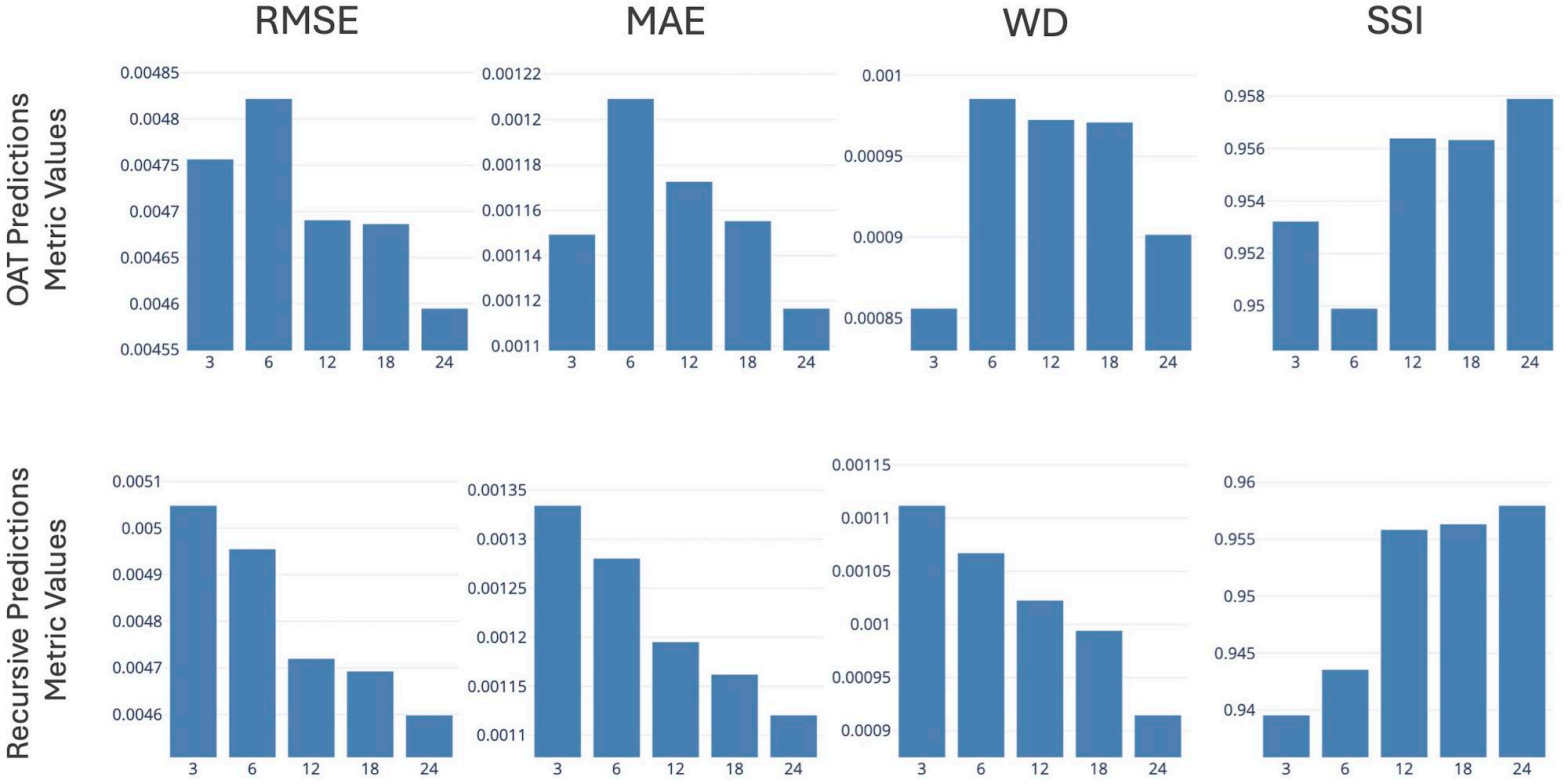
Metric values for one-at-a-time (OAT) and recursive predictions for *G. morhua* with respect to the observed values as functions of lag size (*L*, months) from June 2023 to May 2024 data.

**Figure 6.**
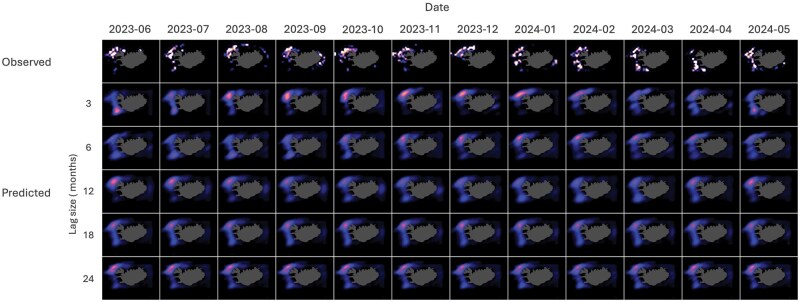
OAT forecast results for *G. morhua* on the test data from June 2023 to May 2024 across varying lag sizes. Higher intensities depict higher probability catch.

**Figure 7.**
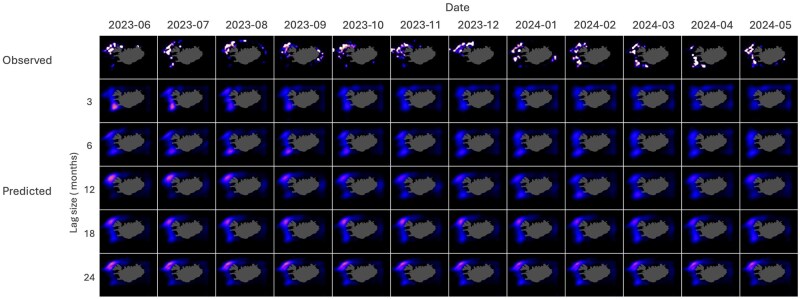
Recursive forecast results for *G. morhua* on the test data from June 2023 to May 2024 across varying lag sizes. Higher intensities depict higher probability catch.

Table 3 and Fig. 5 summarize and illustrate the performance matrix on *G. morhua* test data across varying lag sizes (*L*). A close concurrence is observed between the model’s predictions and the actual observed values as consistently low RMSE and MAE values were observed. Particularly for OAT predictions, the MAE varies from 1.12 × 10^−3^ to 1.21 × 10^−3^ across the different lag sizes, while the RMSE varies from 4.60 × 10^−3^ to 4.82 × 10^−3^. On the other hand, the RMSE and MAE values for recursive predictions range from 4.60 × 10^−3^ to 5.05 × 10^−3^ and 1.12 × 10^−3^ to 1.33 × 10^−3^, respectively. Low WD values, ranging from 0.86 × 10^−3^ to 0.99 × 10^−3^, and high SSI values, ranging from 0.95 to 0.98, were also observed supporting the CATCH model’s robustness and accuracy. This is despite the striking difference between the nature of the observed and the predicted datasets (Figs 6 and 7).

**Table 3.** Specific metric values for OAT and recursive predictions for *G. morhua* with respect to observed values as functions of lag size (*L*) from June 2023 to May 2024 data.

Lag size (*L,* months)	RMSE ×10^3^ (↓)	MAE ×10^3^ (↓)	WD ×10^3^ (↓)	SSI ×10^1^ (↑)
One-at-a-time (OAT)				
3	4.76	1.15	**0.86**	9.53
6	4.82	1.21	0.99	9.50
12	4.69	1.17	0.97	9.56
18	4.69	1.16	0.97	9.56
24	**4.60**	**1.12**	0.90	**9.58**
Mean	4.71	1.16	0.94	9.55
SD	0.08	0.03	0.06	0.03
Recursive				
3	5.05	1.33	1.11	9.40
6	4.96	1.28	1.07	9.44
12	4.72	1.20	1.02	9.56
18	4.69	1.16	0.99	9.56
24	**4.60**	**1.12**	**0.92**	**9.58**
Mean	4.80	1.22	1.02	9.51
SD	0.19	0.09	0.07	0.08

Arrows indicate direction of better scores, i.e. down means the lower the better and up means the higher the better. Bold numbers indicate best performance for each metric across lag sizes. Standard deviations are reported in absolute unscaled units for easier comparison across varying orders of magnitudes.

Figures 6 and 7 show the plotted observed catch (top row) and the predicted probability catch (bottom rows) in months for both OAT and recursive forecasts, respectively. These figures highlight the difference between the nature of the observed and predicted probability catches—with the observed datasets seen as highly discontinuous with multiple local clusters and the predicted datasets as smooth and continuous. The discontinuous nature of the observed dataset is due to the incomplete and uneven sampling from the fishing fleets. The figures also highlight the movements within the data across space and time and how different *L* values affect these movements in the predictions. While general concordance of the predicted with observed data on probability density distributions is apparent for all the lag sizes, movement is more obvious in models with lower *L* values and slowly diminishes as *L* increases.

Tables 4 and 5 present Syrjala’s test *P*-values for both OAT and recursive forecasts across different lag sizes and forecast months. The test evaluates spatial similarity between predicted and observed distributions, with *P*-values greater than .05 indicating no statistically significant difference. In these cases, the model’s predicted spatial patterns can be interpreted as closely matching the observed distributions. However, it is important to note that Syrjala’s test does not confirm distributional equivalence—it only tests for significant difference. Because the test is right-tailed and designed to detect divergence rather than similarity, nonsignificant results suggest spatial agreement but cannot conclusively prove it. On both runs, the lag size *L* = 18 consistently produced the highest average *P*-values (.7255 and .6947 for OAT and recursive predictions, respectively), suggesting that models with this amount of temporal context were most effective at capturing the balance between the spatial structure and the temporal dynamics of observed catch distributions.

**Table 4.** Syrjala’s test *P*-values for different lag sizes (*L* = 3, 6, 12, 18, 24) across 12 forecast horizons for OAT predictions.

Months	Lag size (*L*)				
	3	6	12	18	24
1	0.9708	0.7079	0.8447	0.9960	0.9353
2	0.8309	0.4032	0.4162	0.7602	0.5827
3	1.0000	0.9570	0.8618	0.9999	0.9957
4	0.4262	0.6668	0.8755	0.5343	0.5282
5	0.1545	0.2699	0.1132	0.3878	0.3599
6	0.1002	0.1631	0.0881	0.2733	0.2539
7	0.5728	0.4563	0.5756	0.6241	0.6126
8	0.6497	0.6913	0.8339	0.8420	0.7998
9	0.5663	0.4503	0.6959	0.7337	0.6174
10	0.7440	0.4891	0.8605	0.8048	0.6379
11	0.9421	0.7482	0.9098	0.9074	0.7948
12	0.8406	0.6221	0.8576	0.8427	0.5578
Mean	0.6498	0.5521	0.6611	0.7255	0.6397

**Table 5.** Syrjala’s test *P*-values for different lag sizes (*L* = 3, 6, 12, 18, 24) across 12 forecast horizons for recursive predictions.

Months	Lag size (*L*)				
	3	6	12	18	24
1	0.9674	0.7048	0.8424	0.9945	0.9301
2	0.7965	0.3871	0.4061	0.7618	0.5773
3	0.9979	0.9251	0.8479	0.9995	0.9970
4	0.7536	0.7875	0.8946	0.5339	0.5352
5	0.0973	0.2536	0.0989	0.3768	0.3513
6	0.0214	0.1691	0.0608	0.2558	0.2362
7	0.0803	0.3372	0.4522	0.6010	0.5933
8	0.1251	0.8361	0.6589	0.7960	0.7789
9	0.0572	0.4784	0.4395	0.6813	0.5640
10	0.0439	0.4916	0.5213	0.7316	0.5630
11	0.1037	0.5756	0.6446	0.8761	0.7350
12	0.0186	0.4841	0.2981	0.7274	0.4666
Mean	0.3386	0.5358	0.5138	0.6947	0.6107

**Figure 8.**
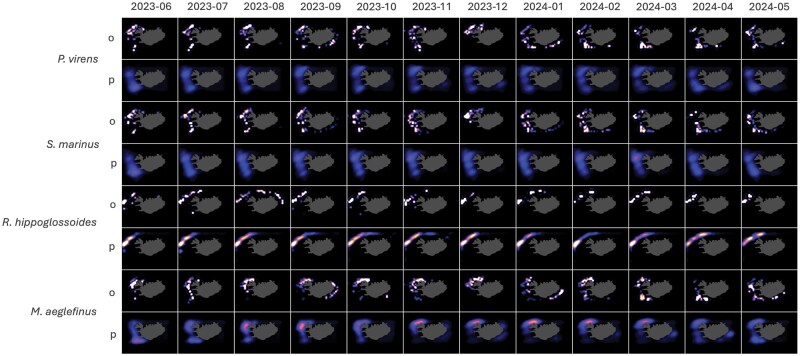
OAT forecast results (*p*) for four species (*P. virens, S. marinus, R. hippoglossoides, and M. aeglefinus*) against the test data (*o*) from June 2023 to May 2024 with lag size of 3 months. Higher intensities depict higher probability values.

Table 6 summarizes performance metrics on the test data from *P. virens, S. marinus, R. hippoglossoides, and M. aeglefinus* with a lag size of 3 months. Similar patterns of close concurrence are observed between the model’s predictions and the actual observed values as consistently low RMSE and MAE values were observed, in the order of 10^−3^. Low WD values, in the order of 10^−3^, and high SSI values, close to unity, were also observed. Figure 8 illustrates the observed and predicted probability densities across time for all the four species. It also highlights the alignment of predictions with the observed data, showcasing a strong correspondence and accurate reflection of overall spatiotemporal patterns despite varying dynamics across species.

**Table 6.** Specific metric values for OAT with respect to observed values across various species (*P. virens, S. marinus, R. hippoglossoides, and M. aeglefinus*) from June 2023 to May 2024 data with lag size of 3 months.

Species	RMSE ×10^3^ (↓)	MAE ×10^3^ (↓)	WD ×10^3^ (↓)	SSI ×10^1^ (↑)
*P. virens*	5.80	1.34	1.20	9.38
*S. marinus*	5.45	1.26	1.07	9.54
*R. hippoglossoides*	7.98	1.19	0.94	9.69
*M. aeglefinus*	5.30	1.22	0.94	9.37
Mean	6.13	1.25	1.04	9.49

Arrows indicate direction of better scores, i.e. down means the lower the better and up means the higher the better.

## Discussion

### Model performance and evaluation

The results of the experiment imply that the CATCH model does a good job at capturing the data’s underlying spatial and temporal patterns. This is exemplified by the favourable performance metrics for the one-at-a-time (OAT) and recursive forecast predictions as shown in Table 3 and Fig. 5. Consistently low RMSE and MAE values, which are in the order of 10^−3^, implies that the model’s predictions closely concur with the actual observations.

The CATCH model’s low WD values, in the order of 10^−3^, also imply that the predicted probability densities closely match the observed distributions, which is crucial for accurate probabilistic forecasting. Despite the difference in the sparsity of the observed and predicted datasets, low WD values show that the model does not overfit and generalizes the patterns well through gradients, not sparse patches, producing predictions that align with ecological intuition rather than overfitting to patches of data. This ensures the model’s capability to extend predictions to unobserved regions, filling data gaps in a plausible manner while ensuring that the predicted distributions maintain structural coherence and environmental relevance. Indeed, this is also supported by the high Structural Similarity Index (SSI) values approaching unity, demonstrating that the model maintains strong structural fidelity in its forecasts across time and signifies that the model preserves important spatial feature structures when compared to the observed data over time. Overall, these results highlight the model’s robustness in capturing both spatial and temporal dynamics while generating predictions that are not only accurate but also ecologically meaningful, making it a valuable tool for potentially understanding and managing fisheries populations.

### Comparison of OAT and recursive forecasts

When comparing the metric values between OAT and recursive predictions, OAT forecasts consistently exhibit lower RMSE, MAE, and WD, along with higher SSI. This highlights the nature of compounding errors in recursive forecasting, as the model gradually uses its own synthetic predictions as input data over time. This is further supported when analysing the results from Syrjala’s test across different forecast horizons, showing a general trend of decreasing performance with increasing recursive forecast months. Additionally, examining the metrics at *L *= 18 reveals that both OAT and recursive predictions starting at this lag size yield values that are close to each other with minimal differences. This trend suggests that as *L* becomes larger, the recursive metrics converge towards those of the OAT predictions until *L* is sufficiently long to project towards the required forecast horizon—one year in this case. This thus implies that at least in the context of the training data used in the study and one year forecast length, utilizing a context length of at least 18 is recommended in order for recursive forecasts to achieve reliability comparable to OAT predictions. Such observations align with literature findings [54, 55] suggesting that long-range recursive forecasting requires a larger context window.

### Effect of lag size

The general trend observed on OAT predictions is that of improving performance as lag size increases. This might indicate that a larger lag size, corresponding to a broader context window and input information, provides the model with a wider context per prediction, enabling more accurate long-term forecasts. However, looking at the trend for the WD metric—which considers the evaluation as a comparison between probability densities—it appears that higher model performance might also be obtained through smaller context windows. This suggests that perhaps two independent temporal patterns are at play, such that increasing or decreasing *L* leads to a trade-off between them. Such is a common scenario in time-series modelling where temporal dependencies are broadly classified into long-term and short-term impacts, typically reflected as trends and fluctuations, respectively [54]. In this case, lower lag values might be more sensitive to short-term dynamics and thus captures them well, despite not reliably capturing long-term relationships. If such a trade-off pattern exists within the dataset used in the study, then the opposite should be true for a model with higher *L*. Results in Fig. 6 provide visual affirmation of this reasoning. As *L* increases, spatial movement and intensity variations of the hotspots become less dynamic. In the observed test data, movement of the hotspots through time from north to south are seen to happen briefly from June to July 2023 and then slowly manifests again from January to May 2024. Such temporal dynamics is seen to be captured only by models with *L* of 3, 6, and 12 with movement diminishing as *L* increases. While increasing lag time improves the metrics and can be beneficial for capturing overarching patterns, it may lead to the attenuation of significant short-term events (e.g. spawning migrations, rapid environmental changes, or regulatory shifts), which are critical in certain forecasting contexts.

### Statistical analysis across forecast horizons

As demonstrated in the previous sections, models equipped with longer temporal context windows, particularly with lag size *L *= 24, consistently outperformed shorter lags in capturing the average spatial structure of observed catch distributions. This trend is reinforced by the results of Syrjala’s test, which show that longer lag models more frequently yield higher, nonsignificant *P*-values, indicating strong spatial congruence between predicted and observed probability density maps. Interestingly, a recurring pattern emerges across both one-at-a-time (OAT) and recursive forecasts: a noticeable dip in *P*-values during months 4 through 7, regardless of the lag size. While most of these values remain above the .05 threshold, implying no statistically significant difference, the consistent drop suggests that the model struggles to replicate spatial dynamics as accurately during this particular window. This may reflect increased variability or complexity in fish movements during this period (e.g. spawning migrations or seasonal transitions), which are less well captured by the model despite longer historical context.

The recursive forecasts provide further insight into the model’s temporal reliability. As predictions are generated from the model’s own outputs rather than ground truth inputs, the impact of error compounding becomes more visible, especially for shorter lag sizes. Notably, models with *L *= 3 and *L *= 6 begin to show performance degradation as early as the fourth month, reflected in declining *P*-values. This suggests that while short-lag models may provide reasonable short-term predictions and finer temporal dynamics, their reliability diminishes over longer horizons, making them less suited for extended multimonth forecasting without correction mechanisms or additional external inputs.

Together, these findings emphasize the importance of lag size selection not only in terms of general accuracy but also in understanding when and where the model’s forecasts are most trustworthy. They also highlight the utility of Syrjala’s test in detecting subtle patterns of forecast deterioration that may not be fully captured by aggregate error metrics alone.

### Multispecies applicability

This phase of the study is guided by a central methodological question: Given the variance in species-specific spatiotemporal patterns, can a unified modelling framework still make accurate forecasts across different species, or is it overly tuned to a narrow behavioural niche, thus limiting its broader applicability? Building on the emphasis on short-term dynamics and considering that the observed data covers only 12 months—where such dynamics are more apparent—the model with *L *= 3 was chosen to predict across the remaining four species (*P. virens*, *S. marinus*, *R. hippoglossoides*, and *M. aeglefinus*). This decision was made because it demonstrated the best performance metrics and visual concordance with respect to capturing the movements within the test data, as evidenced by its performance with *G. morhua.*  Figure 8 and Table 4 present the results of the CATCH model predictions for these species. From the figure, it is evident that the model effectively grasps the spatiotemporal movements shown by the observations, similar to the performance seen in *G. morhua*. The movement of hotspots and intensity variations over time are well represented, indicating the model’s proficiency in modelling essential spatial dynamics for each of the distinct datasets. Quantitatively, this is supported by performance metrics in Table 4 where the values for RMSE, MAE, WD, and SSI fall within similar orders of magnitude as those for *G. morhua*. These consistent metrics across multiple species demonstrate the robustness of the model in capturing the patterns even across varying training data distributions exhibited by different species.

The robustness of the model’s framework suggests that the model potentially possesses the capability to generalize fish species behaviour, at least for bottom-dwelling species that are being represented in this study. Having a single model that can effectively forecast multiple species behaviour is particularly significant, as it implies that the model can capture underlying patterns and movements characteristic of the species as a group. Such generalization capability enhances the model’s utility, potentially reducing the need for species-specific models and extensive amounts of training data for each species, as training can be done using only a handful of representative species to generalize patterns within a species group. This would make the model a valuable tool for predicting distributions of under-represented species in data-limited contexts. Moreover, the model’s proficiency in capturing multispecies interactions could have important implications for ecosystem management and conservation efforts. By accurately modelling and subsequently extracting model explainability insights on multispecies interactions, the CATCH model can provide valuable insights into species distribution patterns, habitat utilization, behavioural correlations, and potential areas of ecological significance. However, it is important to acknowledge potential limitations of the framework used within the study. While the model performs well with the species tested, further validation with additional species and longer observation periods would strengthen confidence in its generalization capabilities. Furthermore, the training and testing data used in the study were not validated in the context of known ecological dynamics from literature sources as the focus was solely the model’s capability to produce accurate forecast given the observations in the data. Future work could thus involve generating and aligning insights with more context on the fish biology of these representative species, testing the model with other species group that exhibit completely different movement patterns, such as pelagic species and training one general model from crafted features that capture multispecies interactions.

### Downstream applications and perspectives

While the CATCH model is designed primarily as a predictive framework for spatiotemporal fish stock probability densities, it is important to acknowledge that practical fishing operations involve real-time decision-making under uncertainty. The data used for model training—drawn from historical fishing activity—reflects areas already frequented by vessels, and as such, the model is inherently conditioned on existing patterns of fishing effort. This limits its ability to explore entirely new fishing grounds and underscores that its outputs should not be interpreted as unbiased estimator of absolute fish biomass across the ocean.

Instead, the value of CATCH lies in its ability to generate adaptive, forward-looking forecasts of relative catch likelihood within known fishing zones. By incorporating dynamic environmental variables that are sensitive to seasonal cycles and long-term climatic trends, the model has potential to learn latent signals of ecological drift. As environmental conditions continue to shift due to climate change, traditional heuristics or static decision rules may become less reliable. In contrast, CATCH offers a mechanism for resilience, enabling skippers and analysts to detect and respond to emerging patterns that differ from historical expectations. In this capacity, CATCH can serve as a diagnostic layer within broader decision-support systems, providing spatial probability distributions that inform questions such as where to initiate fishing operations, when to relocate, and how to anticipate temporal shifts in target species behaviour. These insights can be integrated with operational constraints such as quota status, fleet distribution, and weather forecasts. Importantly, such decision support applications—particularly those involving route planning or real-time adaptation—require robust and temporally aware forecasts of fish distributions. Spatiotemporal prediction is thus not an alternative decision modelling, but a prerequisite for adaptive optimization in uncertain environments.

Future work may explore coupling CATCH’s probabilistic forecasts with decision-making frameworks such as optimal route planning based on reinforcement learning or metaheuristic optimization methods. These methods could simulate skipper behaviour under shifting conditions, translating probabilistic maps into sequential decisions aimed at maximizing catch efficiency or minimizing effort. By positioning CATCH as a modular forecasting engine, this approach aims to bridge the gap between environmental prediction and real-world operational strategy, offering a foundation for advanced data-driven systems in fisheries management.

## Conclusions and recommendations

This study introduced the CATCH model, a deep learning framework utilizing Convolutional-LSTM networks to forecast fish stock probability densities in Icelandic waters. This is the first study in the literature to discuss the utilization of this framework on the previously unexplored data collected by the Icelandic fishing fleet with the hope of aiding the Icelandic fishing industry and environmental sustainability. The model demonstrated high accuracy and robustness in predicting the spatial and temporal distributions of key commercial fish species, as evidenced by low RMSE, MAE, and WD, along with high SSI scores and nonsignificant *P*-values. These metrics show the generalization capability of the model without overfitting and how well it captures intricate spatiotemporal patterns. Interestingly, the model demonstrated potential to generalize across several species, accurately representing the distribution patterns of Greenland halibut, Atlantic cod, haddock, saithe, and golden redfish. This implies that the model might be applied to different species groups, which would eliminate the requirement for species-specific models and make it a useful tool for managing fisheries in situations when data is scarce.

Future recommendations for addressing the limitations and gaps identified within the study may include:

1. *Testing Multispecies Generalization*. Further validation of the model’s generalization capabilities should be conducted by testing it on additional species, including those with different behavioural patterns, such as pelagic species. This will help determine the model’s applicability across diverse species groups. Moreover, the training experiments performed in the current study involved independent CATCH models individually trained on different species and then subsequently used to predict across each of these species. A general model, while more computationally demanding and theoretically more complex, should be trained from a single dataset with crafted features that inherently highlight patterns for multispecies interactions. This could, in turn, forecast distributions even outside the representative species in the training data.
2. *Incorporating Fish Biology Context*. Aligning the model’s insights with detailed fish biology (such as fish length and weight distributions) and ecological data can enhance its predictive power and interpretability. Integrating biological factors such as spawning cycles, feeding habits, and migration patterns could provide a more comprehensive understanding of species distributions.
3. *Enhancing Model Inputs*. Exploring additional environmental/physical variables, such as ocean currents, wind speed, and wind direction, among others, may improve the model’s accuracy and provide deeper insights into the factors influencing fish stock movements.
4. *Long-Term Forecasting*. Extending the model to perform long-term forecasts could be valuable for strategic planning in fisheries management. This would involve training the model with longer time-series data and testing its ability to predict future stock distributions over extended periods.

Enhancing the CATCH model in this way could significantly increase its value as a tool for operational research in the fishing industry by boosting potential profits and increasing the sustainability of fishing operations. Equally important, the model can provide deeper insights into the robustness of individual fish stocks, enabling better strategic measures to ensure sustainability and the preservation of newly spawned generations.

## Data Availability

The data underlying this study are proprietary and were provided by Brim hf., Útgerðarfélag Reykjavíkur hf., Samherji hf., Síldarvinnslan hf., and Guðmundur Runólfsson hf. under strict confidentiality agreements. As such, the data are not publicly available and cannot be shared or accessed at any time, including upon request.
